# Osthole induces G2/M arrest and apoptosis in lung cancer A549 cells by modulating PI3K/Akt pathway

**DOI:** 10.1186/1756-9966-30-33

**Published:** 2011-03-29

**Authors:** Xiaoman Xu, Yi Zhang, Dan Qu, Tingshu Jiang, Shengqi Li

**Affiliations:** 1 Department of Respiratory Medicine, the Shengjing Hospital, China Medical University, Shenyang 110004, PR China; 2 Department of Geriatrics, the Shengjing Hospital, China Medical University, Shenyang 110004, PR China

## Abstract

**Background:**

To explore the effects of Osthole on the proliferation, cell cycle and apoptosis of human lung cancer A549 cells.

**Methods:**

Human lung cancer A549 cells were treated with Osthole at different concentrations. Cell proliferation was measured using the MTT assay. Cell cycle was evaluated using DNA flow cytometry analysis. Induction of apoptosis was determined by flow cytometry and fluorescent microscopy. The expressions of Cyclin B1, p-Cdc2, Bcl-2, Bax, t-Akt and p-Akt were evaluated by Western blotting.

**Results:**

Osthole inhibited the growth of human lung cancer A549 cells by inducing G2/M arrest and apoptosis. Western blotting demonstrated that Osthole down-regulated the expressions of Cyclin B1, p-Cdc2 and Bcl-2 and up-regulated the expressions of Bax in A549 cells. Inhibition of PI3K/Akt signaling pathway was also observed after treating A549 cells with Osthole.

**Conclusions:**

Our findings suggest that Osthole may have a therapeutic application in the treatment of human lung cancer.

## Background

Lung cancer is the leading cause of cancer-related death in the world, and non-small cell lung cancer accounts for approximately 80% of all cases[1,2]. Despite advances in diagnostic and therapeutic, the overall 5-year survival rate in many countries is generally less than 15%[3]. In order to improve the survival rate, intensive efforts have been made to find new anticancer agents, and many attentions have been drawn to herbal medicines owing to their wide range of biological activities, low toxicity and side effects[4-6].

**Figure 1:**
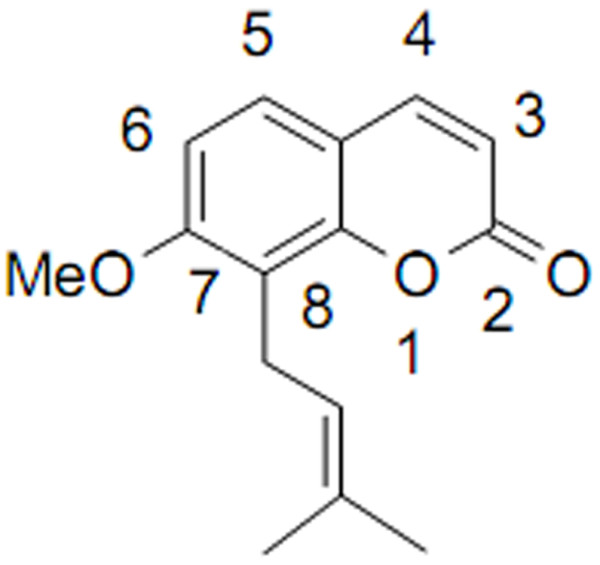
**The structure of Osthole**.

Osthole, 7-methoxy-8-(3-methyl-2-butenyl)coumarin(Figure 1), is an active constituent of *Cnidium monnieri *(L.) Cusson, has been extracted from many medicinal plants such as *Cnidium monnieri *and other plants. Osthole has long been used in traditional Chinese medicine for the treatment of eczema, cutaneous pruritus, trichomonas vaginalis infection, and sexual dysfunction. Recent studies have revealed that Osthole may have antiproliferative[7], vasorelaxant[8], anti-inflammatory[9], antimicrobacterial[10], antiallergic[11], and preventing prophylactic effects in hepatitis[12]. Furthermore, the anticancer effect of Osthole has been reported in few papers[13-17]. These studies have revealed that Osthole inhibited the growth, invasion and metastasis of cancer cells. However, the effects of Osthole on human lung cancer cells remain unclear.

The PI3K/Akt signaling pathway is a critical transduction pathway which plays an important role in regulating cell proliferation, cell cycle and apoptosis[18]. Various types of cancer, including lung cancer, were reported to aberrantly activate this pathway[19]. Recent studies have shown that some anticancer-drugs could induce G2/M arrest accompanying the down-regulation of Akt[20,21]. And the PI3K/Akt pathway participates in the regulation of Bcl-2 family proteins, which are key regulators of the apoptotic pathway[22]. In the present study, we observed that Osthole induces G2/M arrest and apoptosis in lung cancer A549 cells. Osthole-induced G2/M arrest and apoptosis were associated with inhibition of the Cyclin B1, p-Cdc2 and p-Akt expressions, and up-regulation of the ratio of Bax/Bcl-2 proteins.

## Methods

### Reagents

RPMI-1640, trypsin, penicillin and streptomycin were purchased from Biological Industries (Kibutz Beit Haemek, Israel). Fetal bovine serum (FBS) was purchased from Solarbio Science&Technology (Beijing, China). 3-(4, 5-dimethyl thiazol-2yl)-2, 5-diphenyltetrazolium bromide (MTT), dimethyl sulfoxide (DMSO), Propidium iodide (PI), and Hoechst 33342 were purchased from Sigma-Aldrich (St. Louis, USA). Annexin V-FITC and PI double staining kit were purchased from Key Gene (Nanjing, China). Osthole was purchased from the National Institute for the control of Pharmaceutical and biological products (Beijing, China), a 50 mM stock solution of Osthole was dissolved in DMSO and stored at -20°C. Antibodies were purchased from Santa Cruz Biotechnology (Santa Cruz, CA). All other reagents were procured locally.

### Cell line and culture conditions

The human lung cancer cell line A549 was obtained from the China Center for Type Culture Collection (Wuhan, China) and maintained in RPMI-1640 supplemented with 10% FBS, 100 U/ml penicillin, and 100 μg/ml streptomycin at 37°C in a humidified atmosphere of 5% CO2.

### MTT Assay

Cell proliferation was measured using the MTT assay. A549 cells were plated at a density of 1 × 10^4^cells per well in 96-well plates overnight and then treated with different concentrations of Osthole (0, 25, 50, 100, 150, and 200 μM). After 24, 48 and 72 h treatment, 20 μl of MTT solution (2 mg/ml in PBS) were added to each well and the cells were cultured for another 4 h at 37°C. Then the medium was totally removed and 150 μl DMSO was added to solubilize MTT formazan crystals. Finally, the plates were shaken and the optical density was determined at 570 nm (OD570) using a ELISA plate reader (Model 550, Bio-Rad, USA). At least three independent experiments were performed.

### Cell cycle analysis

Cell cycle was evaluated using DNA flow cytometry analysis. A549 cells were plated at a density of 1 × 10^6^cells per well in 6-well plates overnight and then treated with different concentrations of Osthole (0, 50, 100, 150 μM). After 48 h treatment, the cells were harvested and washed twice with PBS, then centrifuged at 1200 ×g for 5 min, fixed in 70% ethanol at 4°C. Before flow cytometry analysis, the cells were washed again with PBS, treated with RNase(50 μg/ml), and stained with PI(100 μg/ml) in the dark for 30 min. The samples were analyzed by FACScan flow cytometer (Becton Dickinson, San Jose, CA).

### Annexin V/PI flow cytometry analysis

Apoptotic rates were determined by flow cytometry analysis using an Annexin V-FITC Apoptosis Kit. A549 cells were plated at a density of 1 × 10^6 ^cells per well in 6-well plates overnight and then treated with different concentrations of Osthole (0, 50, 100, 150 μM) for 48 h. Staining was performed according to the manufacturer's instructions, and flow cytometry was conducted on a FACScan flow cytometer (Becton Dickinson, San Jose, CA). The percentage of the early apoptosis was calculated by annexin V-positivity and PI-negativity, while the percentage of the late apoptosis was calculated by annexin V-positivity and PI-positivity.

### Fluorescent microscopy

A549 cells were treated with different concentrations of Osthole (0, 50, 100, and 150 μM) for 48 h. Cells were washed twice with PBS and fixed with cold methanol and acetic acid (3/1, v/v) before being stained with Hoechst 33342(1 mg/ml) for 30 min at 37°C. Stained cells were observed with a fluorescence microscope(×400, Nikon, Japan).

### Western blotting analysis

The expression of cellular proteins was evaluated by Western blotting. After treatment for 48 h, the cells were washed twice with ice-cold PBS. The total proteins were solubilized and extracted with lysis buffer(20 mM HEPES, pH 7.9, 20% glycerol, 200 mM KCl, 0.5 mM EDTA, 0.5% NP40, 0.5 mM DTT, 1% protease inhibitor cocktail). Protein concentration was determined by bicinchoninic acid (BCA) protein assay. Equal amounts of protein (50 μg) from each sample were subjected to seperate on a SDS-PAGE. After electrophoresis, proteins were electroblotted to polyvinylidene difluoride membranes. The membranes were blocked at room temperature and then incubated at 4°C overnight with the first antibodies of Cyclin B1, p-Cdc2, Bcl-2, Bax, t-Akt and p-Akt seperately. After being washed three times with TBST(20 mM Tris-Cl, pH 7.5, 150 mM NaCl, 1 g/L Tween20), membranes were incubated with secondary antibodies. After incubation, the membranes were washed three times with TBST, and visualization was made using an ECL kit.

### Statistical analysis

The data are expressed as mean ± SD. Statistical correlation of data was checked for significance by ANOVA and Student's *t *test. Differences with P < 0.05 were considered significant. These analyses were performed using SPSS 11.0 software.

## Results

### Osthole inhibited A549 cell proliferation

**Figure 2:**
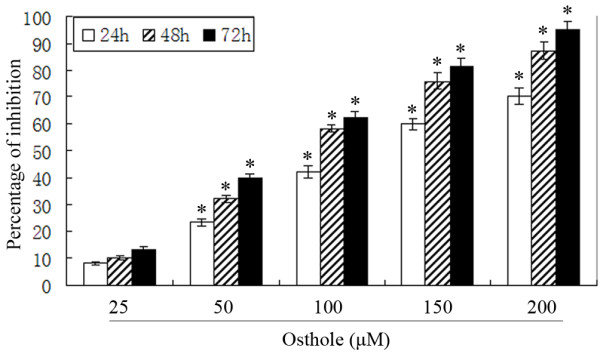
**The proliferative inhibition effects of Osthole on human lung cancer A549 cells**. *p < 0.001 versus control group.

To investigate the growth inhibition effects of Osthole, the cells were treated with different concentrations of Osthole for 24, 48 and 72 h, and the rate of inhibition was determined by MTT assay. We observed that growth of A549 cells was suppressed in a dose- and time-dependent manner(Figure 2).

### Osthole induces G 2/M arrest

**Figure 3:**
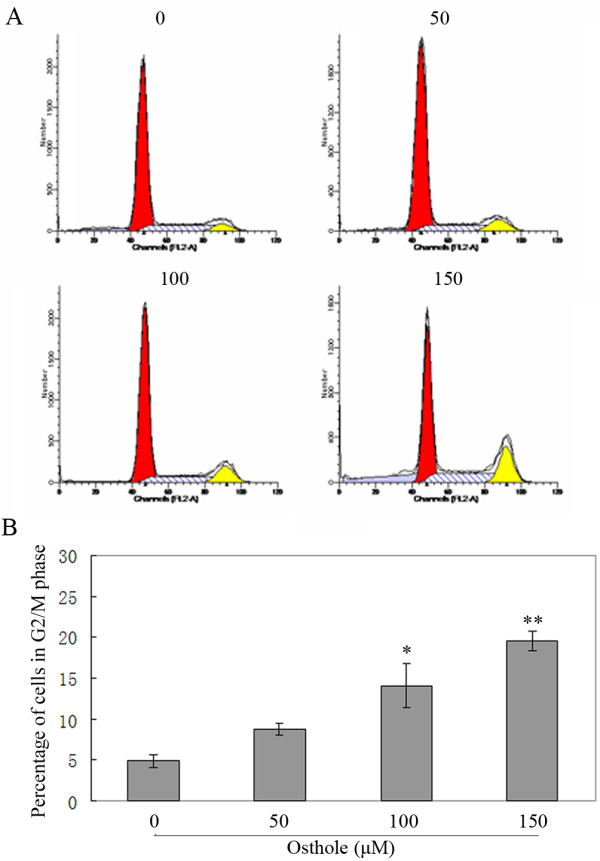
**Cell cycle distribution analysis by DNA flow cytometry**. (A) A549 cells were treated with (0, 50, 100 and 150 μM) Osthole for 48 h. Then the cells were harvested and treated with RNase, stained with PI. The cell cycle distribution was analyzed by flow cytometry. (B) The percentage of cells in G2/M phase in histograms. *p < 0.01, **p < 0.001 versus control group.

To determine whether Osthole inhibits the cell cycle progression of A549 cells, the cells were treated with different concentrations of Osthole (0, 50, 100, and 150 μM) for 48 h and the cell cycle distribution was analyzed by flow cytometry. As shown in Figure 3, the percentage of cells in G2/M phase with Osthole treatment were 4.9%, 8.8%, 14.1% and 19.5% after 48 h, respectively.

### Osthole induces the apoptosis of A549 cells

**Figure 4:**
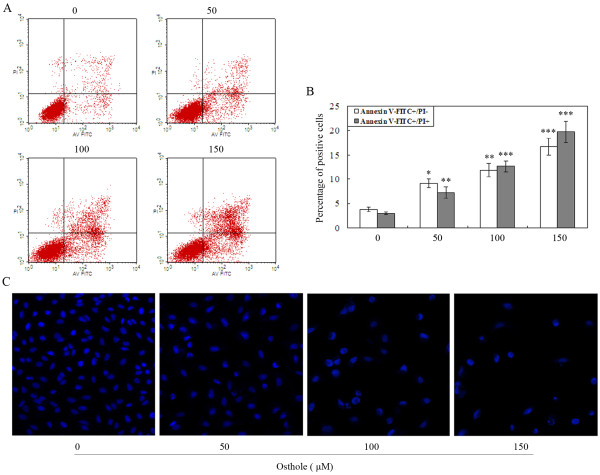
**Apoptosis analysis by flow cytometry and fluorescent microscopy**. (A) Apoptotic rates analysis by Annexin V/PI staining. A549 cells were treated with (0, 50, 100 and 150 μM) Osthole for 48 h. Then the cells were harvested and were stained with Annexin V/PI and flow cytometric analysis was performed to analyze apoptosis rates. (B) Summaries of the apoptosis rates in histograms. *p < 0.05, **p < 0.01, ***p < 0.001 versus control group. (C) Cell apoptosis observed by Hoechst 33342 staining. A549 cells treated with (0, 50, 100 and 150 μM) Osthole for 48 h. Apoptotic cells exhibited chromatin condensations, nuclear fragmentations, and apoptotic bodies.

A549 cells were treated with different concentrations of Osthole (0, 50, 100, and 150 μM) for 48 h and were analyzed by flow cytometry. As showed in Figure 4A, B, the numbers of early and late apoptotic cells were significantly increased compared to control group. The proportion of early and late apoptotic cells in the 150 μM treatment group was about six times higher than in the drug-free group. The proportion of apoptotic cells in treated cells were increased in a dose-dependent manner.

After incubation with different concentrations of Osthole (0, 50, 100, and 150 μM) for 48 h, the cells were examined by fluorescent microscopy analysis. As shown in Figure 4C, condensation of chromatin, nuclear fragmentations and apoptotic bodies were found clearly in treated cells. The results showed that when exposed to Osthole, A549 cells underwent the typical morphologic changes of apoptosis in a dose-dependent manner.

### Osthole decreases Cyclin B1 and p-Cdc2 expressions

**Figure 5:**
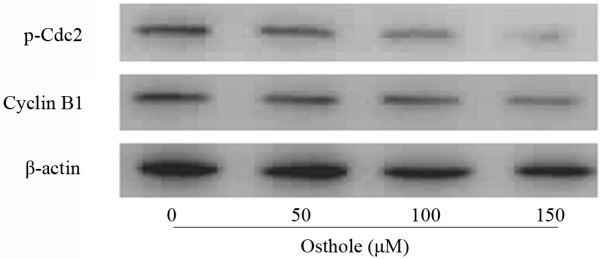
**Effect of Osthole on the expressions of Cyclin B1 and p-Cdc2 by Western blotting analysis**. A549 cells were treated with (0, 50, 100 and 150 μM) Osthole for 48 h. Proteins were extracted, then Cyclin B1, p-Cdc2 and β-actin expressions were analyzed by Western blotting.

To investigate the mechanism underlying cell cycle arrest induced by Osthole, we tested the effect of this compound on p-Cdc2, Cyclin B1 levels. As shown in Figure 5, Western blotting analysis revealed that Osthole decreased the protein levels of Cyclin B1 and p-Cdc2 via a dose-dependent manner.

### Effect of Osthole on expressions of Bcl-2 family proteins

**Figure 6:**
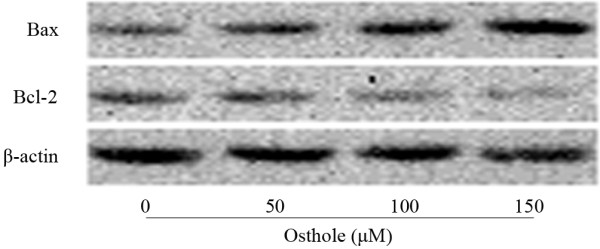
**Effect of Osthole on Bcl-2 family proteins by Western blotting analysis**. A549 cells were treated with (0, 50, 100 and 150 μM) Osthole for 48 h. Proteins were extracted, then Bax, Bcl-2 and β-actin expressions were analyzed by Western blotting.

To investigate the mechanism underlying apoptosis induced by Osthole, we tested the effect of this compound on Bcl-2, Bax levels. As shown in Figure 6, Western blotting analysis revealed that Osthole treatment leads to decrease in Bcl-2 levels and increase in Bax levels as compared to control cells. These results indicated that Osthole up-regulation of the Bax/Bcl-2 ratio in a dose-dependent manner.

### Effects of Osthole on PI3K/Akt pathway

**Figure 7:**
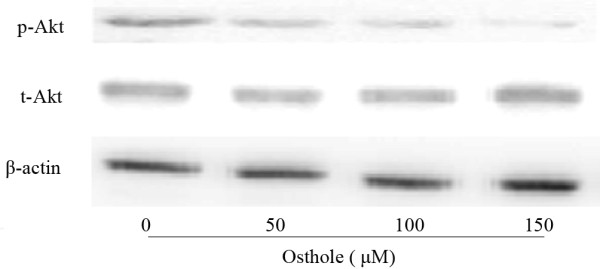
**Effect of Osthole on the PI3K/Akt signaling pathways by Western blotting analysis**. A549 cells were treated with (0, 50, 100 and 150 μM) Osthole for 48 h. Proteins were extracted, then p-Akt, t-Akt and β-actin expressions were analyzed by Western blotting.

In order to better understand the molecular basis of Osthole induced G2/M arrest and apoptosis, we investigated the expression of p-Akt and t-Akt after treatment with Osthole(0, 50, 100, and 150 μM) for 48 h. As shown in Figure 7, the levels of p-Akt are dose-dependently decreased in response to Osthole, while the total Akt protein levels remained constant during Osthole treatment.

## Discussion

Osthole, an active constituent of *Cnidium monnieri *(L.) Cusson, extracted from many medicinal plants and herbs such as *Cnidium monnieri, Angelica pubescens *and some species of *Leguminosae *and *Compositae*. Osthole has been shown to have comprehensive and wider applications as anti-hepatitis, anti-oxidation, anti-inflammatory, anti-microbacterial, and antiallergic effects[7-12]. Furthermore, the anticancer effect of Osthole has been reported in a few papers. Both in vitro and in vivo studies showed that Osthole possessed an anticancer effect by inhibiting human cancer cells growth and inducing apoptosis[13-17]. It is reported recently that Osthole is able to inhibit the migration and invasion of breast cancer cells[15]. Osthole may be a good compound for developing anticancer drugs.

The induction of cell cycle arrest and apoptosis are common mechanisms proposed for the cytotoxic effects of anticancer-drug extracted from herbal medicine[23]. Cell cycle arrest can trigger proliferation inhibition and apoptosis in cancer cells[24,25]. During cell cycle, the G2/M checkpoint is a potential target for cancer therapy. It prevents DNA-damaged cells from entering mitosis and allows for the repair of DNA that was damaged in late S or G 2 phases prior to mitosis[26]. The G2/M checkpoint is controlled by Cdc2 and Cyclin B1[27], and some anticancer-drugs could induce G2/M arrest through down-regulating the expressions of Cyclin B1 and Cdc2[28]. The results in our study showed that treating A549 cells with Osthole resulted in decreased expression of Cdc2 and Cyclin B1, suggesting that decreasing of Cdc2 and Cyclin B1 expression might be the molecular mechanism through which Osthole induced G2/M arrest.

Apoptosis, an important regulator in developmental processes, maintenance of homeostasis and elimination of the damaged cells, is the outcome of a complex interaction between pro- and anti-apoptotic molecules. Proteins of the Bcl-2 family are key regulators of the apoptotic pathway[29,30]. Bcl-2 family can be divided into two subfamilies: one is anti-apoptotic protein such as Bcl-2, the other is pro-apoptotic protein such as Bax. Accumulated data have shown that many anticancer agents induced apoptosis by targeting the proteins of Bcl-2 family and the ratio of Bax/Bcl-2 played a critical role in determining whether cells will undergo apoptosis[31,32]. In our study, by examining the effect of Osthole on Bax and Bcl-2, we found that Osthole increased pro-apoptotic Bax expression and decreased anti-apoptotic Bcl-2 expression, leading to up-regulation of the ratio of Bax/Bcl-2. This might be one of the molecular mechanisms through which Osthole induces apoptosis.

The PI3K/Akt is one of the most important signaling pathways in regulating cell growth, proliferation and apoptosis, and Akt is a major downstream target of PI3K [18]. The PI3K/Akt signaling pathway regulates the development and progression of various cancers by elevating the activity of the anti-apoptotic action of Akt, and the phosphorylation of Akt is routinely used as readout for the Akt activation[33]. In our study, we evaluated the effect of Osthole on the PI3K/Akt pathways by measuring the protein expression levels of total Akt and phospho-Akt protein. We found that treatment of A549 cells with Osthole reduced the protein expression of p-Akt in a dose-dependent manner, while the total Akt protein levels remained constant during Osthole treatment. Recently studies have shown that some anticancer-drugs could induce G2/M arrest and apoptosis accompanying down-regulation of Akt[20-22]. Meanwhile, we also found that Osthole treatment down-regulated Cdc2/Cyclin B1, Bcl-2 protein and up-regulated Bax in our study. In summary, these results indicated that Osthole induced G2/M arrest and apoptosis possibly by down-regulating Akt signaling in human lung cancer A549 cells.

## Conclusions

Our studies demonstrated that Osthole inhibited the growth of human lung cancer A549 cells by inducing G2/M arrest and apoptosis. This might be the important mechanisms of Osthole suppressed the growth of the lung cancer cells. Our findings suggest that Osthole may have a therapeutic application in the treatment of human lung cancer.

## Competing interests

The authors declare that they have no competing interests.

## Authors' contributions

XMX Conceived and the design of the study, carried out the cells studies and drafted the manuscript. YZ carried out the Western blotting studies. DQ participated in cells studies. TSJ performed the statistical analysis. SQL conceived of the study, and participated in its design and coordination. All authors read and approved the final manuscript.
